# Accurate prediction of drug combination risk levels based on relational graph convolutional network and multi-head attention

**DOI:** 10.1186/s12967-024-05372-8

**Published:** 2024-06-16

**Authors:** Shi-Hui He, Lijun Yun, Hai-Cheng Yi

**Affiliations:** 1 School of Information Science and Technology, Yunnan Normal University, Kunming, 650500 China; 2 Engineering Research Center of Computer Vision and Intelligent Control Technology, Department of Education, Kunming, 650500 China; 3 School of Computer Science, Northwestern Polytechnical University, Xi’an, 710129 China

**Keywords:** Drug-drug interaction event, Graph neural network, Multi-head attention, Drug combination

## Abstract

**Background:**

Accurately identifying the risk level of drug combinations is of great significance in investigating the mechanisms of combination medication and adverse reactions. Most existing methods can only predict whether there is an interaction between two drugs, but cannot directly determine their accurate risk level.

**Methods:**

In this study, we propose a multi-class drug combination risk prediction model named AERGCN-DDI, utilizing a relational graph convolutional network with a multi-head attention mechanism. Drug-drug interaction events with varying risk levels are modeled as a heterogeneous information graph. Attribute features of drug nodes and links are learned based on compound chemical structure information. Finally, the AERGCN-DDI model is proposed to predict drug combination risk level based on heterogenous graph neural network and multi-head attention modules.

**Results:**

To evaluate the effectiveness of the proposed method, five-fold cross-validation and ablation study were conducted. Furthermore, we compared its predictive performance with baseline models and other state-of-the-art methods on two benchmark datasets. Empirical studies demonstrated the superior performances of AERGCN-DDI.

**Conclusions:**

AERGCN-DDI emerges as a valuable tool for predicting the risk levels of drug combinations, thereby aiding in clinical medication decision-making, mitigating severe drug side effects, and enhancing patient clinical prognosis.

## Background

Human disease is a major obstacle to human health. Because of the complexity of the disease and the multiple benefits of combination therapy, combination therapy [1] is often used in the treatment of human diseases. For example, multi-drug therapy can reduce the dosage of drugs and improve the therapeutic effect. However, it has been proven that when we take two different drugs at the same time, it may lead to drug effects that do not belong to either of these drugs, that is, drug-drug interactions (DDIs). In recent years, prediction of DDIs has become an important research topic in the field of bioinformatics. Zwart et al*.* found that 28% of all hospitalized patients had at least one potential DDI, with a 1.4% incidence of contraindicated or life-threatening interactions [2]. Mousavi et al. found that the most common type of interaction observed was type C (78.6%), and that this type of interaction does not cause any serious and fatal consequences, meanwhile, 9.2% of patients had type X interactions, which can be harmful and life-threatening [3]. Therefore, there is a practical need to identify the exact risk levels of interaction between drugs. Traditional in vitro and in vivo experiments are time-consuming and labour-intensive [4, 5]. Before the advent of high-throughput technologies [6, 7], one experiment can only detect one single kind of drug-drug interactions. With abundant types of medications available, it is difficult for researchers to one-by-one identifies DDIs through this way, which limits the effectiveness of DDI risk identification. Therefore, computational methods have gained more attention by establishing algorithmic models to predict possible DDI events. These methods are roughly divided into three categories: matrix-based methods, deep learning-based methods, and graph-based methods.

Matrix-based methods typically incorporate background information about a drug into a matrix decomposition, and then similarly calculate drug-to-drug interaction events. Zhang et al. proposed a manifold regularization matrix factorization-based method to predict potential drug interaction events, named MRMF. Manifold regularization based on drug characteristics is introduced into matrix decomposition [8]. Zhang et al*.* use sparse feature learning (SFL) method to project multiple drug features into a common latent (approximate) interaction matrix, and linear neighbourhoods regularization (LNR) based on known drug interaction is introduced to predict DDI events [9]. Yu et al*.* designed a novel model (DDINMF) for DDI prediction based on Nonnegative Matrix Factorization (NMF) [10]. Shi et al*.* developed a unified framework based on three matrix factorization (TMFUF) for predicting DDI events using the side effects of drugs [11]. One issue arises which is that the merging of node domain characteristics cannot be achieved through matrix-based methods.

Over the past few years, deep learning approaches have yielded outstanding results and significant progress in many fields [12–14]. Karim et al*.* proposed to use CNN and LSTM to predict DDI events [15]. Shukla et al*.* propose the integration of convolutional neural network, recurrent neural network and hybrid density networks to predict DDI events [16]. Chen et al*.* introduce a two-layer architecture, including cross-over (based on CNN) and scalar-level modules that can combine internal and external functionality from different granularities [17]. Yi et al*.* proposed a recurrent neural network model featuring multiple attention layers [18]. The deep learning-based methods are more used in Euclidean space data, which is not entirely applicable to drug networks.

On this basis, graph-based model is more suitable for non-Euclidean space data. Arnold K. Nyamabo et al. propose a message-passing neural network in which edges have learnable weights and study molecular structures to predict DDI events [19]. Lin et al*.* propose a knowledge graph neural network (KGNN), an end-to-end framework, which introduces a knowledge graph to predict DDI events by exploring topologies of drugs in the knowledge graph [20]. Feng et al*.* introduce a deep predictor of drug-drug interactions (DPDDI), which uses graph convolution networks (GCN) to learn low-dimensional feature representations and uses a deep neural network (DNN) to train the model [21]. Yu et al*.* propose a SumGNN method consisting of different sub-modules to obtain better aggregate information and perform multi-category prediction [22]. Wang et al*.* proposed a multi-view graphical learning drug embed by designing an end-to-end framework called MIRACLE that included a key-aware messaging network and a GCN encoder [23]. Ma et al*.* proposed using graphic autoencoders to model heterogeneous correlations between different views and target tasks, and adding attention mechanisms to improve interpretability [24].

In most cases, these methods incorporate known DDI networks and multiple biological information, such as 2D and 3D molecular structures [25], interaction profiles [25, 26], targets [9, 15, 25–28], side-effect similarities [15, 25, 26, 28], drug substructure information [9, 26–28], drug enzyme data [9, 15, 26, 27], drug transporter data [15, 26, 28], drug pathways [9, 26–28], SMILES (Simplified Molecular-Input Line-Entry System) sequences [23, 29] and so on.

Recent research has made significant progress in predicting drug-drug interaction events. Systematic reviews reveal the critical role of computational methods in providing support for judicious drug repurposing, extensively applied in the investigation of viral cancers, psoriasis, COVID-19, and specific cancer types such as HPV-related cervical and endometrial cancers [30–36]. Nonetheless, most of these methods still present several limitations. Firstly, they often require accumulating comprehensive and diverse drug attribute information, which can be burdensome for newly emerged drug model prediction. Secondly, the behavioural characteristics of drug nodes in complex network structures are typically underutilized. Most computational models only consider the attributes of drugs themselves, which are employed for simple classification tasks. Thirdly, most existing methods solely aim to predict whether there are adverse effects among proved drug pairs, ignoring the classification of risk levels within different drug combinations. However, it is especially crucial to properly classify levels of risk associated with drug combinations to assist medical staff in making informed drug recommendations.

**Fig. 1:**
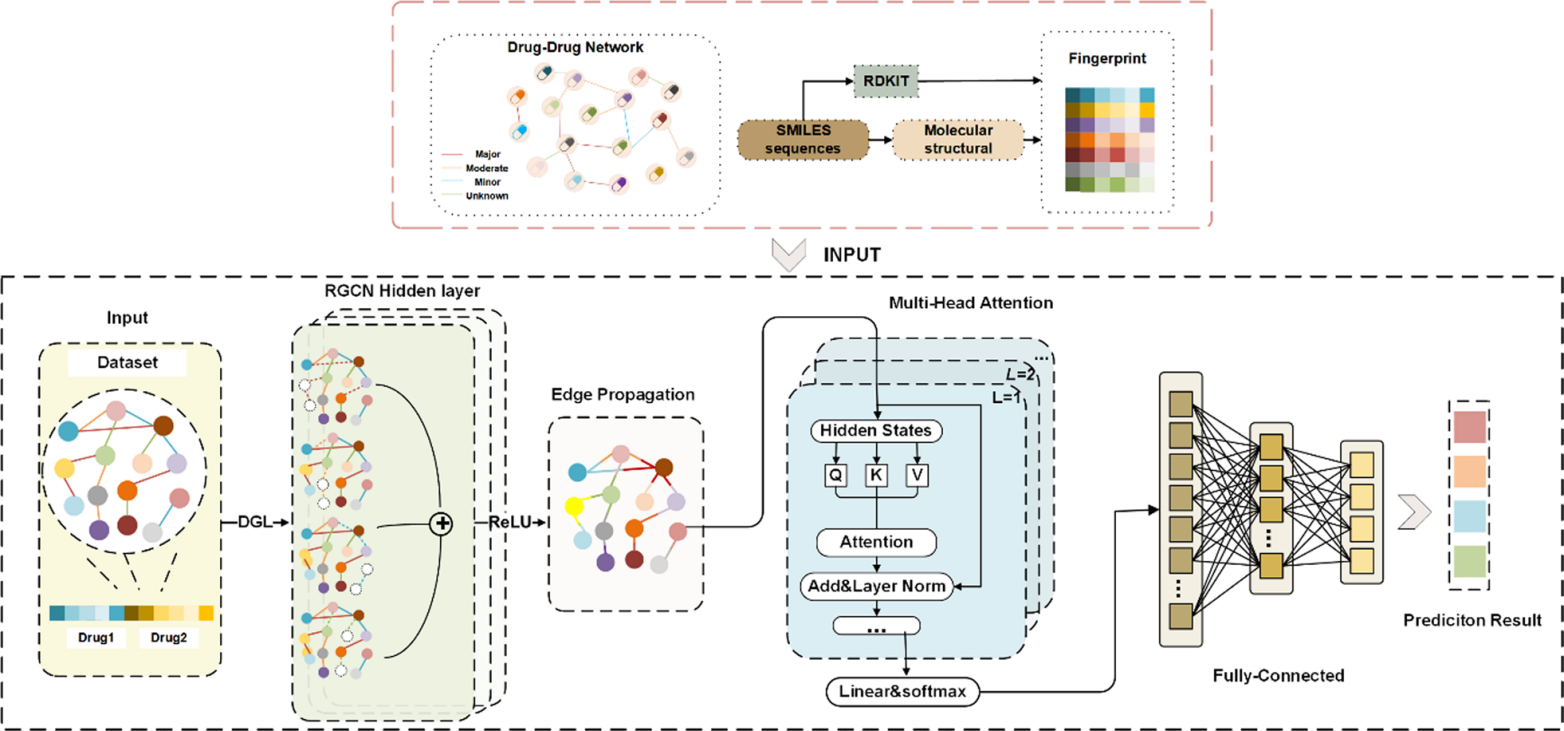
The workflow of the proposed AERGCN-DDI method

In this study, we propose a relational graph convolutional network and multi-head attention-based method to predict risk levels of drug combinations, called AERGCN-DDI. The workflow of AERGCN-DDI is shown in Fig. 1. More specifically, a heterogeneous information graph is constructed by treating drugs as nodes, different risk levels of drug-drug interaction events as edges. Subsequently, the molecule fingerprint generated by the RDKit [37] tool is utilized as node features, and link features are obtained by connecting the features of nodes on both sides. Then, principal component analysis (PCA) is employed to reduce the dimension of the primary attribute features. Finally, a heterogenous graph neural network with multi-head attention modules is proposed to predict DDI events. AERGCN-DDI is tested to predict the combination risk of both approved drugs and newly emerged drug compounds. To evaluate the effectiveness of the proposed method, five-fold cross-validation and ablation study were further conducted. Experimental results demonstrated that AERGCN-DDI can serve as a useful tool for predicting the risk levels of drug combinations, which can help guide clinical medication decisions, reduce serious drug side effects, and improve patient clinical prognosis.

## Methods

### Benchmark datasets

A hierarchical multi-class drug combination dataset was constructed based on the DDinter [38], which contains about 0.24M DDI associations among 1833 approved drugs. Each drug is annotated with basic chemical and pharmacological information and its interaction network. Abundant professional annotations are provided for DDI entries, including severity, mechanism description, strategies for managing potential side effects, alternative medications, etc. The drugs that were unable to obtain compound SMILES descriptors were removed, 1634 drug nodes were ultimately obtained.

**Fig. 2:**
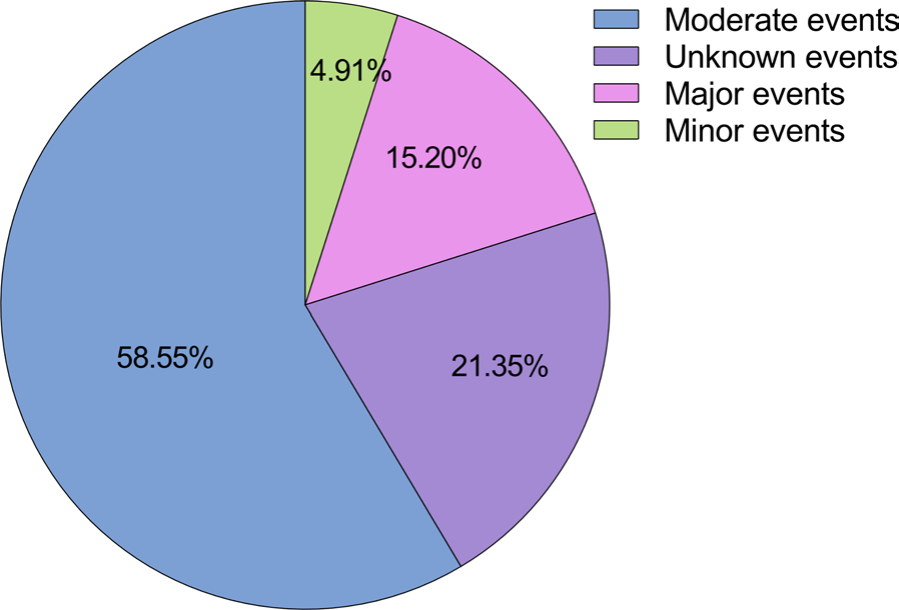
The different risk levels of DDI events in DDInter dataset

The risk level of drug interactions is labeled by senior pharmacists and divided into four levels, including *Major*, *Moderate*, *Minor*, and *Unknown*. *Major* represents life-threatening interactions requiring medical intervention, *Moderate* indicates the interactions that causes disease exacerbation or therapy change, *Minor* means the interactions that limits clinical effects, usually not requiring therapy changes. DDIs lacking mechanism descriptions were classified as '*Unknown*'. Finally, we obtained 221,132 DDI events, of which 47,182 were unknown events, 10,861 were minor events, 129,472 were moderate events, and 33,617 were major events, as shown in Fig. 2.

**Fig. 3:**
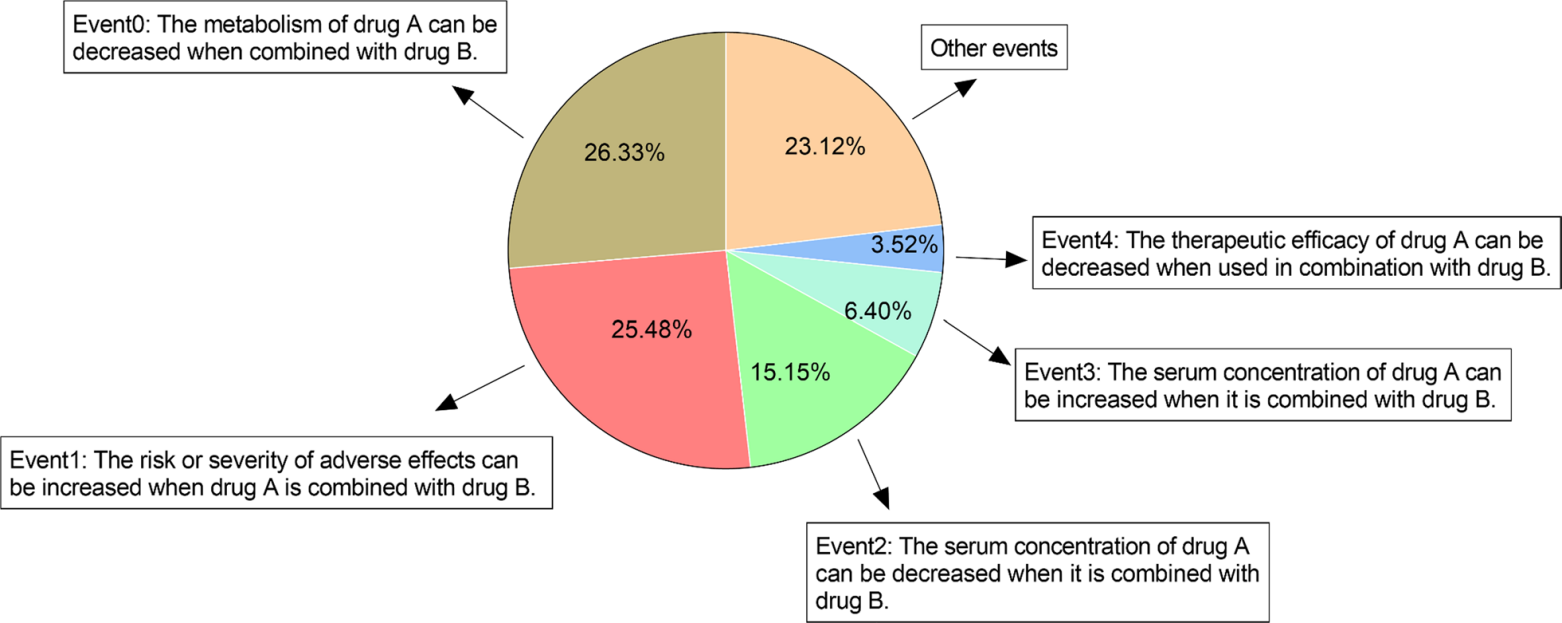
The statistics of DDI events in DrugBank dataset

The second dataset we used was a large-scale drug-drug event dataset constructed by Deng et al. [39] from DrugBank [40], including 572 drugs and 37,264 pair-wise DDIs with DDI types classified into 65 categories. The percentages of all events for this dataset are shown in Fig. 3.

### Construction of heterogeneous information graph

The drug-drug interaction events with different level of risks can be modeled as a heterogeneous information graph, where each node represents a drug, and edges represent different risk levels between drug nodes. Formally, a drug-drug risk rating matrix can be defined as $Y\in {\left(\text{0,1},\text{2,3}\right)}^{\left|{N}_{d}\right|\times \left|{N}_{d}\right|}$, where $\left|{N}_{d}\right|$ denotes the number of drugs. In the matrix, for each entry ${y}_{i,j}=X (i,j\in {N}_{d},i\ne j)$,where $X$ number represents a different risk rating coefficient, and the higher the number of$X$, the higher the risk rating.

In alternative terminology, we can restructure the graph representation of n-array facts from n-array $F=((s,r,o),\left\{\left({a}_{i}:{v}_{v}\right)\right\}{\left(i=1\right)}^{m})$ as a heterogeneous graph $G = (V, E)$. Graphs also are referred to as networks, which assigns nodes to vertices and relationships to $E$. In DDI risk level networks, there are four types of undirected edges between vertices. The vertex set $V$ contains all entities, resulting in $V=\left\{ {V}_{i}, i=\text{1,2},\dots ,n\right\}$ and $E$ is a collection of edges over $V$, ${E}_{ij}=\{\left({V}_{i},{V}_{j}\right),{V}_{i}\in V,{V}_{j}\in V\}$.

### Leveraging molecular fingerprints for drug attribute learning

In order to minimize the reliance on a substantial amount of attribute information, only molecular fingerprint sequences will be employed as drug features. This facilitated the development of lightweight and user-friendly models that align with the practical context of lacking detailed information in the initial stages of new drug development. It is generally assumed that the physical and chemical properties of compounds with similar structures are similar, and similar assumptions are made about their biological activities. This criterion is called Johnson and Maggiora's Law of similarity [21], this is also the basis for computer-aided risk assessment of drug combinations. Molecular fingerprint is a numerical method that can effectively describe the structural information of drug compounds. Previous studies have shown that molecular fingerprints can effectively express the molecular structure of drug compounds. Therefore, we use RDKit [41] to encode of SMILES sequences into Morgan fingerprints as attribute features of drug nodes. In the DDIs link prediction task, the attributes of two different drug nodes with interactive events are concatenated together as edge attributes and input into the model as the first part of the input. And the entire DDI matrix is used as the second part of the input to extract the topology domain information of the DDIs graph.

Furthermore, in order to assess the impact of molecular fingerprint features of different dimensions on the prediction performance, PCA was used to downscale the attribute features into different dimensions. PCA is a widely used dimensionality reduction method. Its main idea is to map $n$-dimensional features to *k*-dimension, which is a new orthogonal feature also called principal component.

1 $$Y = X \cdot P$$

2 $$D = \frac{1}{m}Y^{T} Y = \frac{1}{m}\left( {XP} \right)^{T} \left( {XP} \right) = P^{T} \frac{1}{m}\left( {X^{T} X} \right)P = P^{T} \sum P$$

where *P* is a matrix of $N*K$, which is made up of the column vectors of $K,$ and when $K$ is less than $n$, it is dimensionless [42].

### Enhancing drug combination risk prediction with relational graph convolutional networks

In this section, we introduce a double-layer relational graph convolutional network (RGCN) [43] tailored to capture intricate topology information within DDI graphs. RGCN extends the capabilities of conventional Graph Convolutional Networks (GCNs) by discerning the characteristics of individual relationship types and assigning distinct weight matrices accordingly. Unlike GCNs, RGCNs excel in managing heterogeneous graphs, making them well-suited for DDI networks [44]. The constructed DDI network encompasses four types of edges, with varying weights assigned during model training. The process of updating each node's representation in RGCN involves aggregating information from neighboring nodes. This mechanism enables nodes to glean insights into their topological context while preserving their distinctive characteristics. The propagation model is as follows:

3 $${\mathbf{X}}^{{\left( {l\, + 1} \right)}} \, = \,\sigma \left( {\sum\nolimits_{r\, = \,1}^{R} {\sum\nolimits_{{j \in N_{i}^{r} }} {\frac{1}{{c_{ij} }}{\mathbf{W}}_{r}^{\left( l \right)} \left( {{\mathbf{X}}_{1,j} \, + \,{\mathbf{X}}_{2,j} } \right)} } } \right)$$

Here, ${\mathbf{x}}_{1,j}$ and ${\mathbf{x}}_{2,j}$ are the corresponding components of the feature vectors of node $i$ and $j$. Equation (3) utilizes a double-loop traversal to integrate features from adjacent nodes, thereby fusing them while traversing existing relationships. The output feature of the central node is produced by adding its feature to the aggregated features and applying activation functions. To mitigate overfitting of rare relationships, we introduce two separate methods for regularizing the weights of RGCN layers:

Basis-decomposition:

4 $$W_{r}^{\left( l \right)} = \sum\nolimits_{b\, = \,1}^{B} {a_{rb }^{\left( l \right)} V_{b}^{\left( l \right)} }$$

where ${V}_{b}^{\left(l\right)}\in {R}^{{d}^{\left(l+1\right)}\times {d}^{\left(l\right)}}$ with coefficients ${a}_{rb}^{\left(l\right)}$ such that only the coefficients depend on $r$.

Block-diagonal-decomposition:

5 $$W_{r}^{\left( l \right)} = \oplus_{b = 1}^{B} Q_{br}^{\left( l \right)}$$

where ${W}_{r}^{\left(l\right)}$ consists of block-diagonal matrices, with each

6 $$Q_{br}^{\left( l \right)} \in R^{{\left( {{\raise0.7ex\hbox{${d^{{\left( {l + 1} \right)}} }$} \!\mathord{\left/ {\vphantom {{d^{{\left( {l + 1} \right)}} } B}}\right.\kern-0pt} \!\lower0.7ex\hbox{$B$}}} \right) \times \left( {{\raise0.7ex\hbox{${d^{\left( l \right)} }$} \!\mathord{\left/ {\vphantom {{d^{\left( l \right)} } B}}\right.\kern-0pt} \!\lower0.7ex\hbox{$B$}}} \right)}}$$

contributing to diagonal blocks. For$B=d$, each $Q$ has dimension 1, resulting in ${W}_{r}$ becoming a diagonal matrix. AERGCN-DDI utilized basis-decomposition and have designated the num-bases as multiples of drug pairs risk levels.

### Leveraging multi-head attention for drug interaction prediction

Prediction of newly emerged drugs differs from proved drugs because the former lack interaction information, necessitating models with superior field aggregation capability and stronger predictive performance. This inconsistency prompted us to explore the multi-head self-attention mechanism of transformers as a broad and potent approach to encode knowledge graphs and address the challenge of link prediction.

The update method of the multi-headed attention mechanism is as follows:

7 $${\text{Attention}}\left( {{\mathbf{Q}},{\mathbf{K}},{\mathbf{V}}} \right) = {\text{softmax}}\left( {\frac{{{\mathbf{QK}}^{{\text{T}}} }}{{\sqrt {d_{k} } }}} \right)\left( {{\mathbf{x}}_{1} + {\mathbf{x}}_{2} } \right)$$

where ${\mathbf{x}}_{1}$ and ${\mathbf{x}}_{2}$ are the original feature vectors of two nodes[45].

In our research, we consider unknown relationships as one type of interaction between drugs. After aggregating node and edge features, we generate a set of embedding vectors *Z* for the predicted edges. We apply multi-head attention mechanism to the latent representation sequence *Z* and then score the different types of edges in the classification task. The calculation formula for layer normalization is as follows:

8 $${\text{LayerNorm}}\left( {\mathbf{X}} \right) = \frac{{{\mathbf{X}} - {\mathbf{E}}\left[ {\mathbf{X}} \right]}}{{\sqrt {{\text{Var}}\left[ {\mathbf{X}} \right] + \varepsilon } }} \odot \gamma + \beta$$

### The implementation of the AERGCN-DDI model

The AERGCN-DDI model utilizes a multilayer message-passing mechanism to capture high-order neighboring information. To enhance the prediction of potential DDI events (link prediction), we recalculated the information of nodes and edges. Specifically, the features of edges were generated by combining the features of the edge with those of its two adjacent nodes. The entire model can be descripted as Algorithm 1 below.

**Algorithm 1:**
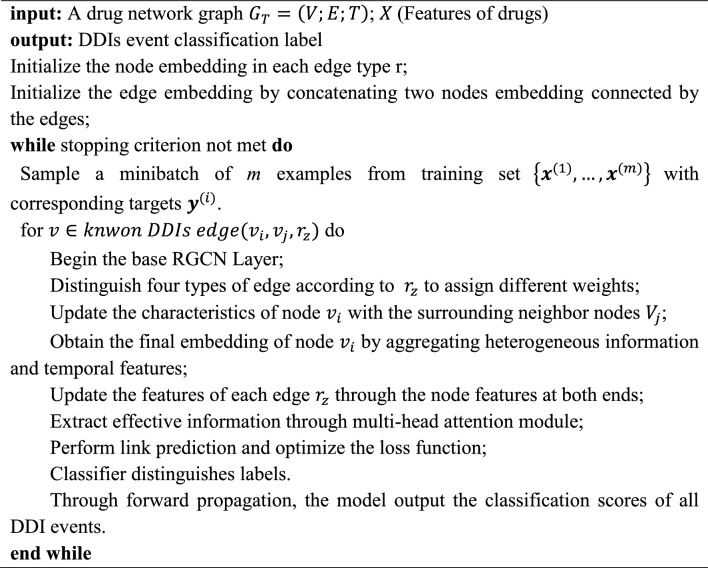
The proposed AERGCN-DDI model.

Let $G(v,\varepsilon )$ be a graph with nodes $v$ and edges $\varepsilon$. The feature for node $v$, and edge ${\left(u,e,v\right)}^{2}$ are represented by ${x}_{v}\in {R}^{{d}_{1}}$ and ${w}_{e}\in {R}^{{d}_{2}}$, respectively. At step $t+1$, the message passing paradigm encompasses node-wise and edge-wise computation [46]:

9 $${\mathbf{Edge - wise}}:m_{e}^{{\left( {t\, + \,1} \right)}} = \emptyset \left( {x_{v}^{\left( t \right)} ,x_{u}^{\left( t \right)} ,W_{e}^{\left( t \right)} } \right),(u,e,v) \in \varepsilon .$$

10 $${\mathbf{Node - wise}}:x_{v}^{{\left( {t\,\, + \,1} \right)}} = \psi \left( {x_{v}^{\left( t \right)} ,\rho \left( {\left\{ {m_{e}^{{\left( {t\, + \,1} \right)}} :\left. {(u,e,v)\, \in \varepsilon } \right\}} \right.} \right)} \right)$$

Here, $\varnothing$ is a message function defined on each edge; The function $\psi$ updates node features by aggregating incoming messages through the reduce function $\rho$.Two-layer RGCN and a multi-head self-attention mechanism are employed to better integrate different types of neighborhood information and capture network structure. Additionally, we utilize AdamW optimizer [47] to train the models by optimizing the cross entropy loss function. The formula of cross-entropy loss is shown as:

11 $$J = - 1/m\mathop \sum \limits_{m} y\log \hat{y}$$

where $y$ is the object, $\widehat{y}$ is the probability of being the object, and $m$ is the number of objects.

### Baseline methods

The graph model achieves network embeddedness by mapping high-dimensional graph data to low-dimensional vectors. To demonstrate the performance and robustness of the proposed AERGCN-DDI, we benchmark a variety of state-of-the-art GNN models, including GCN [48], GAT and GraphSAGE [49], which rely on local domain aggregation of nodes and can be used for link prediction.

**GCN.** The essential purpose of GCN is to extract spatial features of topological graphs. Meanwhile, GCN is a type of neural network layer that operates through inter-layer propagation.

12 $$H^{{\left( {l + 1} \right)}} = \sigma \left( {\tilde{D}^{{ - \frac{1}{2}}} \widetilde{{A{ }}}\tilde{D}^{{ - \frac{1}{2}}} H^{l} W^{l} } \right)$$

where $\widetilde{A }=A+{I}_{N}$,$I$ is the identity matrix. $\widetilde{D}$ is the degree matrix of $\widetilde{A}$,while $H$ is the hidden features of nodes $l$ th layer. $\sigma$ is an activation function that passes information from one layer to the next layer [44].

**GAT.** GAT utilizes a self-attention mechanism to aggregate neighbor nodes, achieving adaptive matching of weights for different neighbors and increasing model accuracy. To make coefficients easily comparable across different nodes, and normalize them across all choices of $j$ using the softmax function:

13 $$a_{ij} = softmax_{j} \left( {e_{ij} } \right) = \frac{{\exp \left( {e_{ij} } \right)}}{{\mathop \sum \nolimits_{{k \in N_{i} }} \exp \left( {e_{ik} } \right){ }}}$$

The attention mechanism is a feedforward neural network with a single layer. Its coefficients can be represented as:

14 $$a_{ij} = { }\frac{{\exp \left( {LeakyReLU\left( {\vec{a}^{T} \left[ {W\vec{h}_{i} |{|}W\overrightarrow {{h{ }}} } \right]{ }} \right){ }} \right){ }}}{{\mathop \sum \nolimits_{{k \in N_{i} }} \exp \left( {LeakyReLU\left( {\vec{a}^{T} \left[ {W\vec{h}_{i} |{|}W\vec{h}} \right]{ }} \right){ }} \right){ }}}$$

**GraphSAGE.** In the GraphSAGE algorithm, each node only samples a portion of its own neighbors to iteratively update its own features. GraphSAGE can use either unsupervised or supervised training. Unsupervised training uses a negative sampling algorithm with the following formula:

15 $$J_{G} \left( {Z_{u} } \right) = - \,{\text{log}}\,\left( {\sigma \left( {Z_{u}^{T} Z_{V} } \right)} \right)\, - \,Q \cdot E_{{v_{n} \sim P_{n} (v)}} \,{\text{log}}\,\left( {\sigma \left( { - Z_{u}^{T} Z_{{V_{n} }} } \right)} \right)$$

Aggregators include: LSTM aggregator, mean aggregator, pooling aggregator, GCN convolution aggregator:

*LSTM aggregator*: LSTM has better feature extraction capabilities, but because there is no obvious sequential relationship between nodes, it is shuffled into the LSTM.

*Mean aggregator*: when aggregating node *V*, compute the average of node *V* and domain eigenvectors:

16 $$h_{v}^{k} \, \leftarrow \,\sigma (W \cdot MEAN\left( {\left\{ {h_{v}^{k - 1} { }} \right\} \cup \left\{ {h_{u}^{k - 1} ,\forall \mu \in N\left( v \right){ }} \right\}{ }} \right)$$

*Pooling aggregator*: In this way, the feature vectors of all the neighbor nodes are passed into a fully connected layer, and then max-pooling aggregation is used:

17 $$h_{v}^{k} \leftarrow \max \left( { \left\{ {\sigma \left( {W_{pool} h_{{u_{i} }}^{k} + b} \right) ,\forall \mu_{i} \in N\left( v \right) } \right\} } \right)$$

**DEML** [50]. Wang et al. proposed an ensemble-based multi-task neural network, for the simultaneous optimization of five synergy regression prediction tasks, synergy classification, and DDI classification tasks. DEML uses chemical and transcriptomics information as inputs. DEML adapts the novel hybrid ensemble layer structure to construct higher order representation using different perspectives. The task-specific fusion layer of DEML joins representations for each task using a gating mechanism.

**DDIMDL** [39]. Deng et al. proposed a multimodal deep learning framework that combines diverse drug features with deep learning to build a model for predicting DDI-associated events. DDIMDL first constructs deep neural network (DNN)-based sub-models, respectively, using four types of drug features: chemical substructures, targets, enzymes and pathways, and then adopts a joint DNN framework to combine the sub-models to learn cross-modality representations of drug–drug pairs and predict DDI events.

**DPSP** [51]. Masumshah et al. introduced a deep learning framework for predicting multiple drug side effects, divided into two steps. Firstly, it collects various drug information that may affect Drug-Drug Interactions (DDIs), such as individual drug side effects, targets, enzymes, chemical substructures, and pathways, to construct novel features. Then, predictions of 65, 100, and 185 categories of DDI events in DS1, DS2, and DS3 are executed through a deep multimodal framework.

**GADNN** [52]. Nejati M et al. proposed a method to predict DDIs by considering the influence of different drug-related features. Their approach consists of two stages. In the first stage, four basic drug datasets are used to generate embedding vectors for each drug separately. Next, a new graph attention mechanism dynamically calculates the contribution coefficient of each dataset, and the weighted combination of these vectors is used to predict drug-drug interactions probability through a dense neural network.

### Experiment setup and evaluation metrics

To evaluate the performance of the proposed method, five-fold cross-validation is first conducted. The whole benchmark dataset is randomly divided into five subsets, one-fold is employed as test set each time, while the remaining four sets are employed as training data, cycle five times and take the average result as final result. To accomplish the task of predicting DDIs between unknown (newly emerged) drugs, we adopt a new data partitioning method. We divided the dataset into two major groups: confirmed (proved) drug categories and novel (newly emerged) drug categories. The latter refers to drugs that lack any prior data and thus, any relevant relationships were removed from the dataset. Based on the partitioned dataset, we divide the corresponding DDI dataset into between confirmed drug pairs, confirmed drug-novel drug pairs, and novel drug pairs. Our model is trained on confirmed drug pairs dataset and performs prediction tasks on confirmed drug pairs (Task 1), confirmed drug-novel drug pairs (Task 2), and novel drug pairs (Task 3), respectively. The final average results of these operations can explain the stability of the proposed model.

Six indicators are adopted to measure the multi classification performance of the model, including accuracy (Acc), Area Under the Precision-Recall Curve (AUPR), Area Under the Receiver Operating Characteristic Curve (AUC), F1 score, Precision and Recall with AUPR and F1 are more sensitive to severe imbalances data. Micro metrics are used for AUPR and AUC, while macro metrics are used for other measurements. The definitions of these indicators can be described as follows:

18 $$Acc = \frac{TP + TN}{{TN + TP + FN + FP}}$$

19 $$precision = \frac{TP}{{TP + FP}}$$

20 $$Recall = \frac{TP}{{TP + FN }}$$

21 $$F1 = \frac{2 \times precision \times recall}{{precision + recall}}$$

where the *TN*, *PN*, *FN* and *FP* denote the number of correctly predicted positive and negative samples, wrongly predicted positive and negative samples, respectively. In addition, we use the *Micro* mode to calculate AUC and Recall, which treats each element of the label indicator matrix as a label. In contrast, F1 calculates each label in a *Macro* mode and finds their unweighted average.

## Results and discussion

**Fig. 4:**
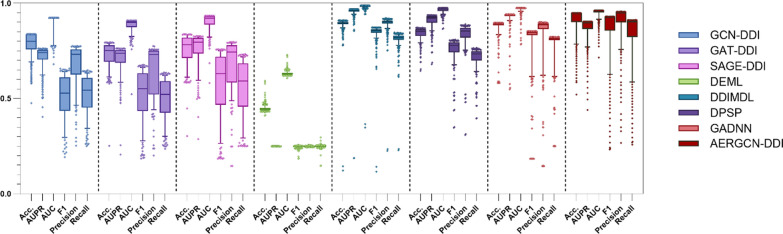
The ACC, AUPR, AUC, F1, Precision and Recall of compared methods on Task 1 of the DDInter dataset

**Fig. 5:**
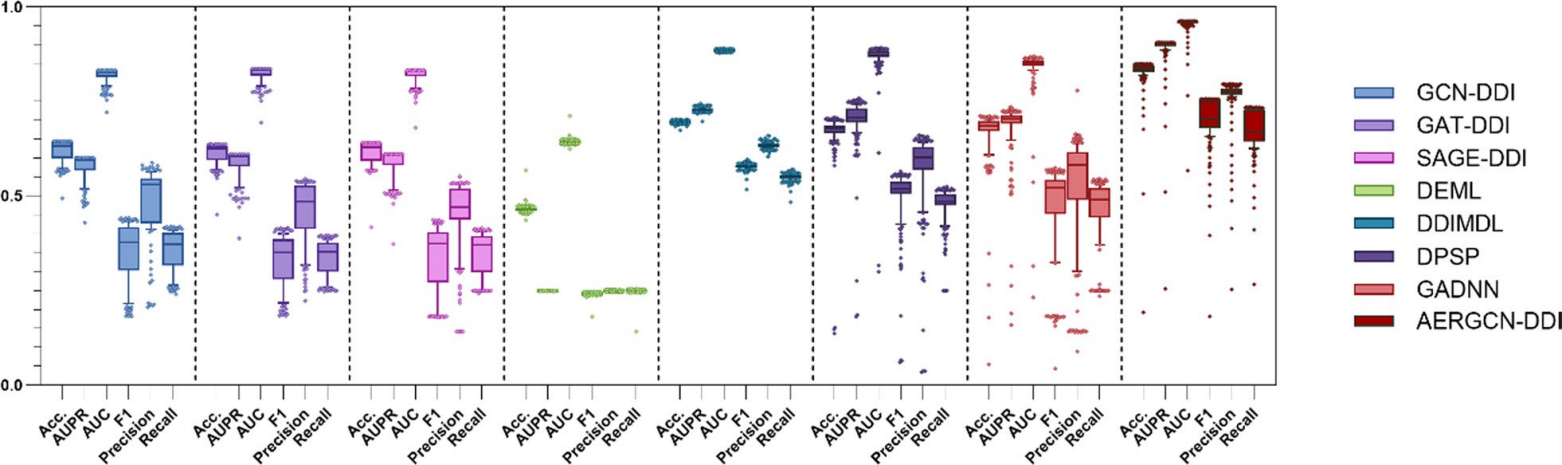
The ACC, AUPR, AUC, F1, Precision and Recall of compared methods on Task 2 of the DDInter dataset

**Fig. 6:**
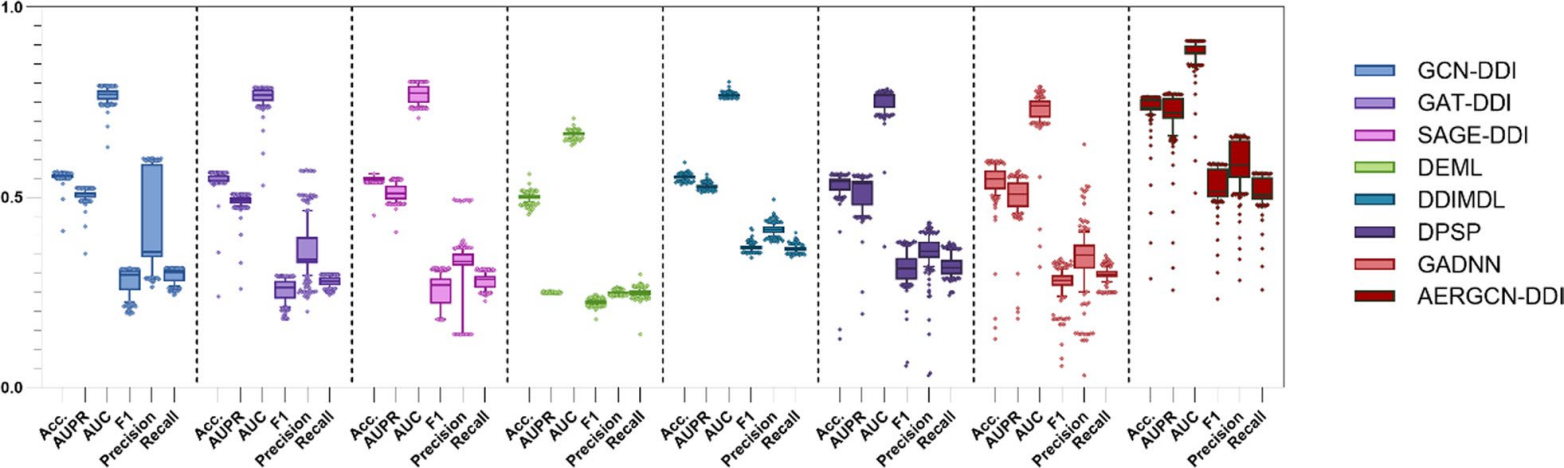
The ACC, AUPR, AUC, F1, Precision and Recall of compared methods on Task 3 of the DDInter dataset

To evaluate the performance of the AERGCN model, we conducted extensive experiments on three tasks, comparing AERGCN with seven state-of-the-art methods under fivefold cross-validation. Tables 1, 2, 3, Figs. 4, 5, and 6 present the performance of the comparison models, including GCN-DDI, GAT-DDI, SAGE-DDI, DEML, DDIMDL, DPSP, GADNN, and AERGCN-DDI.

**Table 1:** The performance of AERGCN-DDI on Task 1 of the DDInter dataset

Method	ACC	AUPR	AUC	F1	Precision	Recall
GCN-DDI	0.8477 ± 0.0274	0.7969 ± 0.0213	0.9213 ± 0.0065	0.7742 ± 0.049	0.8263 ± 0.0299	0.7396 ± 0.0553
GAT-DDI	0.8421 ± 0.0276	0.8166 ± 0.0342	0.9274 ± 0.0111	0.7689 ± 0.0508	0.7935 ± 0.0212	0.7531 ± 0.067
SAGE-DDI	0.9102 ± 0.0461	0.8812 ± 0.0328	0.9547 ± 0.0123	0.8715 ± 0.0689	0.8945 ± 0.066	0.8522 ± 0.0704
DEML	0.5866 ± 0.008	0.2501 ± 0.0002	0.7244 ± 0.0053	0.192 ± 0.0056	0.2502 ± 0.0005	0.1889 ± 0.023
DDIMDL	0.9003 ± 0.0081	**0.9625 ± 0.006**	**0.9855 ± 0.0023**	0.8593 ± 0.0109	0.9074 ± 0.0069	0.8236 ± 0.0141
DPSP	0.8687 ± 0.003	0.9364 ± 0.0011	0.9752 ± 0.0006	0.803 ± 0.0033	0.8788 ± 0.0036	0.756 ± 0.0035
GADNN	0.8963 ± 0.001	0.9362 ± 0.0021	0.9743 ± 0.0009	0.8524 ± 0.0021	0.8964 ± 0.0024	0.8184 ± 0.0025
**AERGCN-DDI**	**0.9381 ± 0.015**	0.901 ± 0.0047	0.9615 ± 0.0011	**0.9148 ± 0.0209**	**0.9317 ± 0.0286**	**0.9004 ± 0.0141**

Bold indicates the method that performs best on this indicator

**Table 2:** The performance of AERGCN-DDI on Task 2 of the DDInter dataset

Method	ACC	AUPR	AUC	F1	Precision	Recall
GCN-DDI	0.6391 ± 0.0072	0.6095 ± 0.0114	0.8328 ± 0.0078	0.4168 ± 0.0183	0.5326 ± 0.0193	0.3986 ± 0.0151
GAT-DDI	0.6319 ± 0.0067	0.6131 ± 0.0063	0.8332 ± 0.0043	0.3957 ± 0.0156	0.5334 ± 0.0186	0.3809 ± 0.0124
SAGE-DDI	0.643 ± 0.01	0.6241 ± 0.0269	0.8362 ± 0.0172	0.4473 ± 0.0371	0.5335 ± 0.013	0.4251 ± 0.0293
DEML	0.5829 ± 0.0138	0.25 ± 0.0002	0.7219 ± 0.0092	0.1886 ± 0.0073	0.2501 ± 0.0005	0.178 ± 0.028
DDIMDL	0.7092 ± 0.0113	0.739 ± 0.0162	0.8866 ± 0.0077	0.5806 ± 0.0214	0.6608 ± 0.0324	0.5418 ± 0.0221
DPSP	0.6866 ± 0.0153	0.7214 ± 0.0253	0.8783 ± 0.0107	0.535 ± 0.0262	0.6202 ± 0.0352	0.4992 ± 0.0241
GADNN	0.6954 ± 0.0107	0.7081 ± 0.0056	0.8557 ± 0.005	0.5478 ± 0.0101	0.6174 ± 0.0268	0.52 ± 0.0089
**AERGCN-DDI**	**0.8493 ± 0.0029**	**0.8893 ± 0.0111**	**0.9573 ± 0.0027**	**0.7596 ± 0.0088**	**0.8086 ± 0.0096**	**0.7286 ± 0.0148**

Bold indicates the method that performs best on this indicator

**Table 3:** The performance of AERGCN-DDI on Task 3 of the DDInter dataset

Method	ACC	AUPR	AUC	F1	Precision	Recall
GCN-DDI	0.5586 ± 0.0205	0.5146 ± 0.0138	0.7716 ± 0.0123	0.2726 ± 0.0344	0.371 ± 0.1327	0.2937 ± 0.0184
GAT-DDI	0.5364 ± 0.0265	0.4811 ± 0.0291	0.7404 ± 0.0215	0.2861 ± 0.0241	0.352 ± 0.0432	0.2944 ± 0.0138
SAGE-DDI	0.5614 ± 0.0136	0.5248 ± 0.026	0.7818 ± 0.0136	0.2073 ± 0.0343	0.307 ± 0.0636	0.2629 ± 0.0187
DEML	0.5661 ± 0.0163	0.2502 ± 0.0002	0.7107 ± 0.0109	0.19 ± 0.0074	0.2504 ± 0.0006	0.1976 ± 0.0431
DDIMDL	0.5512 ± 0.0317	0.5297 ± 0.0467	0.7655 ± 0.0263	0.3403 ± 0.0347	0.4045 ± 0.0394	0.3388 ± 0.0301
DPSP	0.5398 ± 0.0159	0.5221 ± 0.0367	0.7608 ± 0.0211	0.3266 ± 0.036	0.375 ± 0.0269	0.3251 ± 0.0279
GADNN	0.5729 ± 0.0212	0.5286 ± 0.0369	0.751 ± 0.0242	0.2691 ± 0.0508	0.3224 ± 0.1184	0.2954 ± 0.0264
**AERGCN-DDI**	**0.7266 ± 0.317**	**0.7566 ± 0.0211**	**0.9012 ± 0.0102**	**0.5686 ± 0.0531**	**0.687 ± 0.0455**	**0.5396 ± 0.0597**

Bold indicates the method that performs best on this indicator

### Comparison of AERGCN-DDI and comparative methods on Task 1

To evaluate the effectiveness of our method for drug-drug interaction extraction in a hot-start environment (Task 1), we compared the comparative effectiveness of AERGCN with seven other state-of-the-art models. The experimental results are shown in Table 1. From the experimental results, we conclude that the AERGCN-DDI model achieves the best performance in predicting proven drug-drug interaction events under warm-start conditions, and its performances on ACC, AUPR, AUC, F1, Precision, and Recall are 93.81%, 90.1%, 96.15%, 91.48%, respectively, 93.17%, and 90.04%. Of these, ACC, F1, Precision, and Recall all achieved optimal performance, improving over the suboptimal methods by 2.79%, 4.33%, 3.53%, and 4.82%, respectively. To examine the overall effectiveness of the various methods in more detail, we present in Fig. 4 the performance of all the baseline models for all the events in ACC, AUPR, AUC, F1, Precision, and Recall statistical boxplots. These results demonstrate the excellent performance of the AERGCN-DDI method in the task of drug interaction prediction. Relatively speaking, our proposed AERGCN-DDI model performs the best in predicting the interactions between proved drugs in terms of their effectiveness.

### The performance of AERGCN-DDI on Task 2 and Task 3 under five-fold cross-validation

To validate the experimental performance of the proposed model in a cold-start environment, we simulated the scenario of new drug emergence and performed a five-fold cross-validation. In Task 2, we simulated the interaction prediction of old and new drugs, and in Task 3, we simulated the interaction prediction of new and new drugs. The complete and detailed experimental results are shown in Tables 2 and 3, while Figs. 5 and 6 provide a visual presentation of the relevant data.

In Task 2 and Task 3, AERGCN-DDI showed significant advantages in all evaluation metrics. In Task 2, it outperforms the suboptimal method by 14.01%, 16.79%, 7.07%, 17.9%, 14.78%, and 18.68% in terms of ACC, AUPR, AUC, F1, Precision, and Recall, respectively. In Task 3, AERGCN-DDI outperforms the suboptimal method by 15.37%, 22.69%, 11.94%, 22.83%, 28.25% and 20.08%. This indicates that AERGCN has stronger predictive ability and generalization when facing the scenario of emergence of new drugs, and is more suitable for potential relationship mining of unknown drugs, which provides strong support for further research and application in the field of drug interaction prediction.

**Fig. 7:**
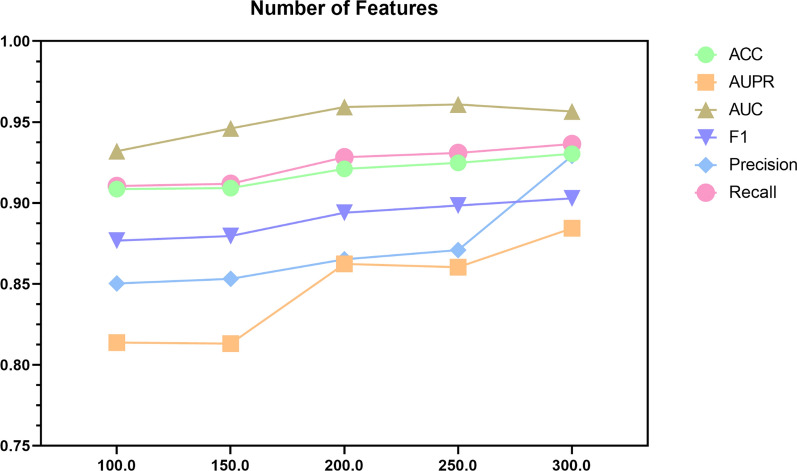
The performance of AERGCN-DDI under different feature dimensions

To verify the effect of different embedding dimensions on the experimental results, we introduce PCA to generate 100, 150, 200, 250, and 300 dimensional feature dimensions and input them into the AERGCN-DDI Model (Task 1), the experiment results show that the 300-dimensional feature can obtain the best value. Figure 7 shows the results of AERGCN-DDI with various numbers of embedding dimensions, Notably, as we increase the number of embedding dimensions, the evaluation indicators of the training and testing sets steadily increase, so the feature dimension is set to 300.

### Comparison of AERGCN-DDI and other state-of-the-art methods on the DrugBank dataset

To further validate the effectiveness of AERGCN-DDI in the multi-classification scenario of DDI events, we utilized DrugBank dataset which consists of 65 classes and is characterized by imbalanced data. To highlight the outstanding performance of our model, we compared AERGCN with the following state-of-the-art DDI prediction methods. Of note, the data for our comparative models are derived from the experimental results presented in the MSEDDI article:

**DeepDDI** [53] consists of SSP and DNN. It takes chemical structures and drug names as inputs and generates human-readable sentences that describe the DDI types.

**Lee’s method** [54] proposed employs autoencoders and a deep feed-forward network, which are trained with SSP, GSP, and TSP of known drug pairs, to predict the pharmacological effects of DDIs.

**DDIMDL** [39] employs four drug features: chemical substructures, targets, enzymes, and pathways. It uses a joint DNN framework to combine the sub-models, learn cross-modality representations of drug pairs, and predict DDI events.

**MDF-SA-DDI** [55] combines two drugs in four different ways and inputs the resulting drug features into four different drug fusion networks (Siamese network, convolutional neural network, and two autoencoders) to obtain potential feature vectors for drug pairs. Then, potential feature fusion is performed using self-attention mechanisms.

**MSEDDI** [56] designs three-channel networks to handle biomedical network-based knowledge graph embedding, SMILES sequence-based notation embedding, and molecular graph-based chemical structure embedding. These channels' output features are then combined through a self-attention mechanism.

As shown in Table 4, on DrugBank dataset, our method is superior to contrast methods. AERGCN-DDI achieves the best performance with a high accuracy of 58.34%**,** and improved the accuracy by 13.83%, the AUPR by13.88%, the AUC by 0.76% than Suboptimal method. The comparison with other state-of-the-art methods on the dataset 2 further reveals the advantages of our proposed AERGCN-DDI in predicting muti-types DDI events. The evaluation results comprehensively demonstrate the promising performance and broad prospects of AERGCN-DDI.

**Table 4:** The performance of all methods on DrugBank dataset

Method	ACC	AUPR	AUC	F1
DeepDDI	0.3602	0.2781	0.9059	0.1373
Lee’s method	0.4097	0.3184	0.8302	0.2022
DDIMDL	0.4075	0.3635	0.9512	0.1590
MDF-SA-DDI	0.4378	0.3810	0.8675	**0.2326**
MSEDDI	0.4451	0.3999	0.9543	0.1691
**AERGCN-DDI**	**0.5834**	**0.5387**	**0.9619**	0.0911

Bold indicates the method that performs best on this indicator

### Ablation study

To validate the effectiveness of using drug fingerprints as node attributes and to verify the efficiency of different components in AERGCN-DDI, including the multi-head attention mechanism and edge propagation module, we performed ablation experiments. The following are the different variants utilized for ablation experiments:

${\varvec{A}}{\varvec{E}}{\varvec{R}}{\varvec{G}}{\varvec{C}}{{\varvec{N}}}_{{\varvec{w}}/{\varvec{o}}\boldsymbol{ }\boldsymbol{ }{\varvec{F}}{\varvec{P}}}$: This is a variant of the AERGCN-DDI model that does not use the node fingerprint feature, but only the topology information in the DDI network.

${\varvec{A}}{\varvec{E}}{\varvec{R}}{\varvec{G}}{\varvec{C}}{{\varvec{N}}}_{{\varvec{w}}/{\varvec{o}}\boldsymbol{ }\boldsymbol{ }{\varvec{A}}{\varvec{T}}}$: It is the original AERGCN-DDI model without the addition of the multi-head attention component.

${\varvec{A}}{\varvec{E}}{\varvec{R}}{\varvec{G}}{\varvec{C}}{{\varvec{N}}}_{{\varvec{w}}/{\varvec{o}}\boldsymbol{ }\boldsymbol{ }{\varvec{E}}{\varvec{P}}}$: It is the original AERGCN-DDI model without the addition of the edge propagation component.

According to the analysis in Table 5, AERGCN-DDI performs significantly better than the other variant models on all tasks and assessment metrics. On the contrary, the variant model without the fingerprint feature exhibited the significantly lowest performance. Specifically, $AERGC{N}_{w/o FP}$ with the molecular fingerprint removed showed the most significant decrease in effectiveness in Task 1, with decreases in ACC, AUPR, AUC, and F1 of 0.3454, 0.3946, 0.1994, and 0.7522, respectively, and $AERGC{N}_{w/o EP}$, with the side propagation module removed, also performed only better than $AERGC{N}_{w/o FP}$ in Tasks 2 and 3. In Task 3, $AERGC{N}_{w/o AT}$'s ACC (0.7362) was slightly higher than ACC of AERGCN-DDI (0.7266), but the performance on AUPR, AUC, and F1 was reduced by 0.2087, 0.0889, and 0.0062. In conclusion, the model performance can be made better by effectively integrating and utilizing different modules, including drug fingerprinting, multi-attention mechanism, and edge propagation components.

**Table 5:** The ablation performance of AERGCN-DDI on different tasks

Task	Method	ACC	AUPR	AUC	F1	RECALL
Task1	$\text{AE}RGCN-DDI$	**0.9381**	**0.901**	**0.9615**	**0.9148**	**0.9004**
	$AERGC{N}_{w/o FP}$	0.5927	0.5064	0.7621	0.1861	0.1482
Task2	$AERGCN-DDI$	**0.8493**	**0.8893**	**0.9573**	**0.7596**	0.7286
	$AERGC{N}_{w/o FP}$	0.5678	0.6193	0.7446	0.1811	0.2500
	$AERGC{N}_{w/o AE}$	0.8295	0.7309	0.9117	0.6914	0.7717
	$AERGC{N}_{w/o EP}$	0.7189	0.6517	0.8759	0.5596	0.6931
Task3	$AERGCN-DDI$	0.7266	**0.7566**	**0.9012**	**0.5686**	0.5396
	$AERGC{N}_{w/o FP}$	0.5615	0.5710	0.6801	0.1798	0.1404
	$AERGC{N}_{w/o AT}$	0.7362	0.5479	0.8123	0.5624	0.6060
	$AERGC{N}_{w/o EP}$	0.5606	0.4790	0.7432	0.2586	0.5409

Bold indicates the method that performs best on this indicator

The experimental results show that drug fingerprints as node properties are most important features of AERGCN-DDI. Drug fingerprints provide rich information about the structure and properties of drug molecules, which helps the model to better understand drug interactions and effects. The experimental results show that the performance of the variant model lacking drug fingerprint features is significantly reduced, further validating the importance of drug fingerprints in the model.

Furthermore, the edge propagation module is one of the key components of the AERGCN-DDI model, which helps the model to better utilize the edge attribute information, including the mode of action and effects of drug combinations. The results of the ablation experiments show that the performance of the variant model with the edge propagation module removed significantly decreases, further confirming the importance of the edge propagation module in the model.

Lastly, the multiple attention mechanism is another key component of the AERGCN-DDI model. This mechanism allows the model to simultaneously focus on different drug interaction features, thus improving the model's ability to capture complex interactions. In the ablation experiments, the performance of the variant model with the multi-head attention mechanism removed decreased in Task 2 and Task 3, indicating that the multi-head attention mechanism plays an important role in enhancing the model performance.

In summary, the drug fingerprint as a node attribute, edge propagation module, and multi-head attention mechanism are key components of the predictive performance of AERGCN-DDI. Their effective integration and utilization enable the AERGCN-DDI model to predict drug-drug interactions more accurately, providing important support for drug development and clinical applications.

## Conclusions

In this work, we proposed a novel approach, the AERGCN-DDI model, which leverages relational graph convolutional networks (RGCN) and multi-head attention mechanisms to predict the specific risk levels associated with drug combinations. Our model utilizes RGCN to comprehend the topological and semantic characteristics of drug nodes, distinguishing between four distinct risk levels and aggregating diverse domain information. Additionally, the incorporation of multi-attention mechanisms enhances our model's capability to capture multi-level topology information effectively. In contrast to conventional experimental setups, we conducted experiments tailored to simulate the emergence of new drugs in real-world scenarios, where these drugs have no prior interactions with existing ones. Our DDI prediction task achieved remarkable accuracy rates, with 93.81% for established drugs, 84.93% for newly introduced drugs, and 72.66% when both drugs were novel. This shows that our model exhibits excellent performance in both warm-start and cold-start environments. In addition, we performed cross-dataset validation, especially after using the DrugBank dataset for validation, to further validate the reliability and applicability of our model. Also, we conducted ablation experiments to validate the importance of each component module in the model. The limitation of the model is that the dataset of the proposed model may be biased towards common drug interactions, while the ability to generalize to rare drug interactions is limited. In future work, in order to enhance the applicability and robustness of the AERGCN-DDI model, it is recommended to integrate more drug features such as molecular structure or pharmacokinetics. Also, exploring different graph structures or incorporating temporal information into the model architecture may improve its performance. In addition, applying the model to predict interactions other than drug-drug interactions (DDIs), such as drug-disease interactions or drug-food interactions, could help to extend its application in clinical practice. The proposed AERGCN-DDI model has proved to be an efficient and competitive drug combination risk prediction tool, to aid in medical decision-making, drug development, and disease treatment, yielding better and safer medical interventions and services.

## Data Availability

The code and datasets are freely available at: https://github.com/ShiHHe/AERGCN-DDI.
